# Dental professionals’ views on motivational interviewing for the prevention of dental caries with adolescents in central Norway

**DOI:** 10.1186/s12903-023-03649-w

**Published:** 2023-11-20

**Authors:** Eva Lassemo, Helen D. Rodd, Marit Slåttelid Skeie, Jan-Are K. Johnsen, Hege Nermo, Kari Sand, Randi Krog Eftedal, Tone Natland Fagerhaug, Arefe Jasbi, Zoe Marshman, Göran Dahllöf, Marikken Høiseth

**Affiliations:** 1 Department of Health Research, SINTEF Digital, SINTEF, Trondheim, Norway; 2 School of Clinical Dentistry, University of Sheffield, Sheffield, UK; 3 Department of Clinical Dentistry, University of Bergen, Bergen, Norway; 4 Center for Oral Health Services and Research, Mid-Norway (TkMidt), Trondheim, Norway; 5 Department of Clinical Dentistry, Faculty of Health Sciences, UiT The Arctic University of Norway, Tromsø, Norway; 6 Department of Public Health and Nursing, Norwegian University of Science and Technology (NTNU), Trondheim, Norway; 7 Department of Design, Faculty of Architecture and Design, Norwegian University of Science and Technology (NTNU), Trondheim, Norway; 8 Department of Dental Medicine, Division of Orthodontics and Paediatric Dentistry, Karolinska Institutet, Stockholm, Sweden

**Keywords:** Prevention, Promotion, Oral health, Motivational interviewing, Adolescents

## Abstract

**Background:**

Establishing positive oral health behaviours during adolescence should be a key priority to improve lifelong oral health. However, changing adolescent behaviours is known to be a challenge. Motivational interviewing (MI) is a method of working with patients to activate their motivation for change and has shown promising results within the dental setting. Yet, little is known about the actual experiences and perspectives of Norwegian dental health professionals in delivering motivational interviewing as part of routine care to their young patients. The overall aim of the present study was to explore the implementation of motivational interviewing by dentists and dental hygienists, employed by the Norwegian Public Dental Service, for their adolescent patients.

**Methods:**

As part of the larger #Care4YoungTeeth <3 project, a Norwegian Research Council funded four-year Collaborative Project to Meet Societal and Industry-related Challenges, an online survey was developed and administered to dental personnel (n = 168) in one region of Central Norway. Data were analysed by descriptive statistics and two-sample tests of proportions at the 95% confidence level.

**Results:**

A total of 98 dental personnel responded to the survey (response rate 58.3%), of which 37 were dental hygienists (response rate 72.5%) and 61 were dentists (response rate 52.1%). A greater proportion of hygienists reported implementing this intervention compared to dentists (78.4% versus 50.8%; p = 0.007). Similarly, a greater proportion of hygienists (83.8%) stated that they had received training in MI compared to dentists (65.6%; p = 0.051). About 80% of dentists and 90% of dental hygienists felt that they understood the principles of MI. However, only about 45% and 60%, respectively, felt confident in its use. Dental hygienists found MI more usable in their work (p = 0.052), to a greater extent want to use MI (p = 0.002) and found that using MI works well (p < 0.001), as compared to dentists.

**Conclusions:**

A high proportion of dental professionals working within a Norwegian public dental service have received training in MI. However, barriers to implementation for adolescent patients and differences in practice between dentists and hygienists warrant further enquiry.

## Background

Oral diseases are among the most prevalent diseases globally and continue to constitute a significant public health challenge [1]. The most prevalent and preventable oral disease is dental caries; a recent meta-analysis based on European studies among 12–19-year-olds reported an overall caries prevalence (including enamel caries) of 77% [2]. The determinants of oral disease are complex, including social, environmental, behavioural, and physiological factors acting in concert [3].

Adolescence is a critical phase in determining lifelong oral health as this period coincides with the eruption of the full permanent dentition. Establishing positive oral health behaviours during these years is opportune and a good investment for improved oral health-related quality of life and a reduction in the future risk of oral diseases and subsequent treatment burden [4]. However, changing adolescent behaviours has acknowledged challenges, and interventions aimed to change oral health habits in this young population may have limited success [5–7]. A systematic review of approaches used to prevent dental caries within a general dental practice setting, conducted by Kay, Vascott [8], suggested that behaviour change is key to improving oral health.

Motivational interviewing (MI) [9] is one method of working with patients to activate their motivation for change and can be defined as a “collaborative, person-centered form of guiding to elicit and strengthen motivation for change” [10], (p. 137). As opposed to being a “technique”, MI is presented as a simple communication method requiring a complex set of skills used in a flexible way to adjust to client feedback [10]. Professional training in MI involves acquiring knowledge and skills connected to its four main principles [11]: (1) expressing empathy, seeking to develop a good rapport with the client, increase perceived understanding, reduce potential tendency of change resistance, and explore inner thoughts and motivations; (2) developing discrepancy, which entails seeing reasons why the client should change based on identifying a gap between their values and current adverse behaviours; (3) rolling with resistance, respecting reluctance to change from the client as common rather than irrational conduct; and (4) supporting the client’s self-efficacy, pursuing recognition that confidence in one’s own ability to change is critical for successful change efforts.

Since its more widespread adoption in the early 1990s, MI has been used to promote healthy behaviours across a broad range of areas such as exercise, heavy alcohol use, smoking, gambling, and oral health [12, 13]. A growing number of systematic reviews have evaluated the delivery of MI, specifically for adolescents and young adults, in a variety of settings. Schaefer and Kavookjian [14] concluded that MI was efficacious in improving adherence and reducing symptoms in adolescents with chronic illnesses, notably diabetes, asthma and HIV. MI has also been found to reduce excess alcohol intake in adolescents with problematic substance use [15]. In contrast, the evidence-base for MI to reduce drug use in adolescents remains equivocal [15, 16].

A review of the evidence for MI in general dental practice has concluded that it does have potential for helping adult patients with poor oral health [17]. However, while in some studies it has been demonstrated that MI is effective in guiding patients to change oral health-related behaviours such as snacking and tooth brushing [18], other studies have found inconclusive evidence for the effectiveness of MI to improve oral health behaviours [19]. In the context of oral health in early childhood, research on the use of MI to support parents to change their child’s early risk status has shown that MI significantly impacts on dental visits for fluoride varnish and oral health knowledge, but for other behaviours such as frequency of toothbrushing or the frequency of use of sweets as a reward the effect of MI remains inconclusive [20].

While the general success of MI in improving oral health has been variable [21], in adolescents, MI has reportedly been more effective than traditional dental health education in eliciting positive changes in oral health behaviours and thus preventing dental caries [18, 22]. Furthermore, improvements achieved through MI have been found to be sustained over 12–24 months [18, 22].

When considering the implementation and appropriateness of MI within dental practice, it is paramount to understand the context of the healthcare system and service providers. With respect to the present study, it should be noted that the Norwegian Public Dental Service (PDS) provides free dental care for children (0–18 years of age) [23]. Furthermore, professional guidelines for child and adolescent dental services in Norway highlight MI as the preferred method for oral health behaviour change [24]. This recommendation was based on evidence from several systematic reviews [12, 21, 25] which have demonstrated better outcomes such as caries reduction, improved dental attendance and improved diet when using MI compared to conventional methods, i.e., traditional health education focusing on disseminating information and giving normative advice.

To align with the expectation that MI is routinely embedded within the Norwegian PDS, all educational institutions with programmes in dentistry and/or dental hygiene in Norway teach MI at varying depth, from lectures alone to lectures combined with courses, role-play, and supervised training in clinical practice. Notably, MI is promoted within the dental hygiene curriculum as a tool to support smoking cessation, as this is integral to managing periodontal disease, as well as reducing the risk of oral cancers [26, 27]. Although education and policy appear to support the use of MI in Norway, little is known about the actual experiences and perspectives of Norwegian dental health professionals in delivering MI as part of routine care to their young patients [28, 29].

Therefore, the overall aim of the present study was to explore the implementation of MI by PDS employed dental health professionals for their adolescent patients. The specific objectives were to: (1) investigate reported use of MI, (2) determine the training received in this approach, (3) gain insight into experiences of MI delivery and (4) compare responses from dental hygienists and dentists.

## Materials and methods

The present study is part of the larger #Care4YoungTeeth <3 project, a Norwegian Research Council funded four-year Collaborative Project to Meet Societal and Industry-related Challenges. The overarching aim of #Care4YoungTeeth <3 is to contribute to improving the oral health of all adolescents, regardless of social, geographical, or economic background. All details related to participant selection and data collection were reported to the Norwegian Centre for Research Data (currently a part of Norwegian Agency for Shared Services in Education and Research) with reference number 346,466. As this survey was carried out anonymously and contained no personal data, it was not subject to any further assessment or approval. Consent was provided by completing and submitting the survey.

A web-based anonymous questionnaire was developed in Nettskjema, provided by the University of Oslo, by an expert group of researchers and clinical practitioners within the fields of dentistry and health services research. The MI-related items were partly based on a survey originally developed by researchers at the University of New Mexico to study the effectiveness of a MI training protocol [30] and work focusing on the use of MI by health personnel [31] and partly developed in-house based on the aim and scope in the main project #Care4YoungTeeth <3 [32]. In total eight items were used from the New Mexico evaluation, three of which were included in the analyses for the present paper. These three items were all concerned with adolescent motivation for change and not context dependent. The items not included for analyses were all concerned with practitioner use of MI.

The content and wording of the questionnaire was piloted by seven dentists and dental hygienists in the Trøndelag PDS, and amendments were made based on their comments and recommendations. In this study, the term *adolescent* referred to 12- to 18-year-olds. In addition to background information (five items), the questionnaire consisted of four main parts: (1) prevention of oral disease in adolescence (15–20 items); (2) managing oral health care of adolescents (12 items); (3) training in MI (4–8 items) with a subsection on the general use of MI (18–25 items); and (4) use of MI when working with adolescents (1–14 items). Respondents were directed through the questionnaire based on their answers, such that the total number of items answered could vary between 39 and 86. The questionnaire ended with two open-ended questions allowing respondents to give more detailed feedback on their use of MI within adolescent dental care. This present paper will report on background information and items relating to training (part 3) and management of adolescents (part 4).

The response format included both closed questions, allowing for one (where options were mutually exclusive such as whether the respondent has received training in use of MI) or more than one answer (where multiple options were possible such as what mode of training in the use of MI the respondent had received), and a five-point Likert scale responding on statements regarding the use of MI where responses ranged from strongly disagreeing to strongly agreeing, with the additional option of ‘don’t know’. The estimated time to complete the questionnaire was 10–15 min.

Data collection was done in collaboration with Trøndelag PDS and carried out in the period from April to August 2022. The survey was sent to all dentists and dental hygienists in Trøndelag PDS. The Trøndelag county director for dental health informed his employees in advance about the survey, stressing that participation was voluntary. In total four reminders were sent. No incentives for completing the survey were offered.

The survey was distributed to 168 dental professionals, of which 51 were dental hygienists and 117 dentists. A total of 98 dental personnel responded to the survey (response rate 58.3%). Of the 98 respondents, 37 were dental hygienists (response rate 72.5%) and 61 were dentists (response rate 52.1%).

Data were analysed using STATA, StataCorp, USA. In comparing responses from dental hygienists and dentists, two-sample tests of proportions at the 95% confidence level were applied.

## Results

### Characteristics of the participants

With regard to age, about 60% of dental hygienists and 65% of dentists were under the age of 40, which is representative of the professions in the region. The dentists and dental hygienists had, on average, worked in clinical practice for 12 years (median = 10; range = 1–37) and 15 years (median = 12; range = 1–39), respectively. Nearly 90% of dental hygienists and 38% of dentists reported that more than half of their patient population consisted of 18 years or younger. In total, 89% (n = 33) of the dental hygienists and 82% (n = 50) of the dentists, reported having approximately the same number of patients under the age of 12 as in the group 12- to 18 years.

### Reported use of MI

Overall, 61.2% (n = 60) of respondents reported using MI with their adolescent patients; with a greater proportion of hygienists implementing this intervention compared to dentists (78.4% versus 50.8%, p = 0.007). As shown in Table 1, those dental health professionals who adopted MI used it for a variety of clinical situations, depending on the specific needs of their patients. The most common indication for MI use, by both professional groups, was in relation to changing adolescents’ oral hygiene practices (96.8% and 100% of dentists and dental hygienists respectively using MI). Both groups also commonly applied MI when giving dietary or smoking/snuff cessation advice. However, MI was less frequently used to address non-attendance amongst adolescent patients (38.7% and 37.9% of dentists and dental hygienists at all using MI, respectively).

**Table 1:** Reported use of MI by dental professionals, for adolescent patients, according to clinical need. With % of respondents reporting overall use

Reported use of MI	Overall (n = 98)	Dental hygienists (n = 37)	Dentists (n = 61)	Dental hygienists vs. dentists (p value)
Overall use with adolescent patients	60 (61.2%)	29 (78.4%)	31 (50.8%)	p = 0.007
As part of oral hygiene instruction	59 (98.3%)	29 (100%)	30 (96.8%)	p = 0.331
As part of dietary advice	52 (86.7%)	27 (93.1%)	25 (80.7%)	p = 0.158
Snuff/smoking cessation	39 (65.0%)	18 (62.1%)	21 (67.7%)	p = 0.650
Management of non-attendance	23 (38.3%)	11 (37.9%)	12 (38.7%)	p = 0.949
Other applications	2 (2.0%)	1 (3.4%)	1 (3.2%)	p = 0.966

Of the respondents, n = 39 (40%), of which n = 19 (51%) dental hygienists and n = 20 (33%) dentists, confirmed being aware of the national guidelines on using MI while working with adolescent patients, including learning resources for MI. However, only 10 (25%), of which n = 6 (32%) dental hygienists and n = 4 (20%) dentists, reported regularly using the guidelines.

In total, 60.2% (n = 59) of respondents, 81.1% (n = 30) and 47.5% (n = 29) of dental hygienists and dentists, respectively, reported using MI in their work. Respondents who reported using MI overall (not only with adolescents) in their clinical work were asked to describe their follow-up regimen for patients who had received this intervention. No follow-up of MI was reported by 24.1% (n = 7) and 20% (n = 6) dentists and dental hygienists, respectively. However, the majority of MI users reported follow-up (reinforcement) of this intervention at the patient’s next routine dental recall visit (62.1% and 66.8% of dentist and dental hygienists, respectively).

A small proportion of dental hygienists, 10.8% (n = 4), and a somewhat higher proportion of dentists, 36.1% (n = 22), stated that they did not use MI in their clinical practice. The most common reason for non-adoption was their lack of training in this intervention (50% of dentists and 75% of dental hygienists). Other reported barriers to MI use included: the perceived length of time that it took (as stated by 36.4% of dentists) and its complexity (9% of dentists and 25% of dental hygienists). A small proportion of dentists (13.6%) stated that they did not feel that the intervention was of value, a view which was supported by individual comments of MI seeming ‘fake’ or ‘unnecessary’. Some non-adopters of MI (22.7% of dentists and 25% of dental hygienists) expressed a preference for using other behavioural approaches to manage their young patients.

### Training in MI

Overall, 72.5% (n = 71) reported that they had previously received training in the use of MI. Significantly more dental hygienists 83.8% (n = 31) compared to 65.6% (n = 40) of dentists (p = 0.051) had received training. However, there was considerable disparity in the extent and recency of this training, although no significant differences between professions (Table 2). Almost half of both professional groups recalled receiving MI-related teaching within their undergraduate curriculum (40% of dentists and 48.4% of dental hygienists). However, a larger proportion of respondents reported completion of postgraduate training in MI (72.5% of dentists and 67.7% of dental hygienists). A small number of participants (5% of dentists and 9.7% of dental hygienists) had undertaken self-directed learning in the field of MI. In terms of recency of training, more than half of both dental hygienists (58.1%, n = 18) and dentists (57.5%, n = 23) most recently received MI training 6–10 years ago.

**Table 2:** Mode of and recency of MI training received by dental professionals

MI training experience	Overall (n = 98)	Dental hygienists (n = 37)	Dentists (n = 61)	Dental hygienists vs. dentists (p value)
Has had MI training	71 (72.5%)	31 (83.8%)	40 (65.6%)	p = 0.051
Mode of training (of those having received training)				
In undergraduate education	31 (43.7%)	15 (48.4%)	16 (40%)	p = 0.479
Postgraduate education course	50 (70.4%)	21 (67.7%)	29 (72.5%)	p = 0.660
Self-directed learning	5 (7.0%)	3 (9.7%)	2 (5%)	p = 0.443
Recency of training				
Within the last 2 years (2021-)	7 (9.9%)	2 (6.5%)	5 (12.5%)	p = 0.400
Within 3–5 years ago (2018–2020)	13 (18.3%)	6 (19.4%)	7 (17.5%)	p = 0.837
Within 6–10 year ago (2013–2017)	41 (57.7%)	18 (58.1%)	23 (57.5%)	p = 0.960
More than 10 years ago (before 2013)	10 (14.1%)	5 (16.1%)	5 (12.5%)	p = 0.665

### Dental professionals’ attitudes to the use of MI for adolescent patients

**Fig. 1:**
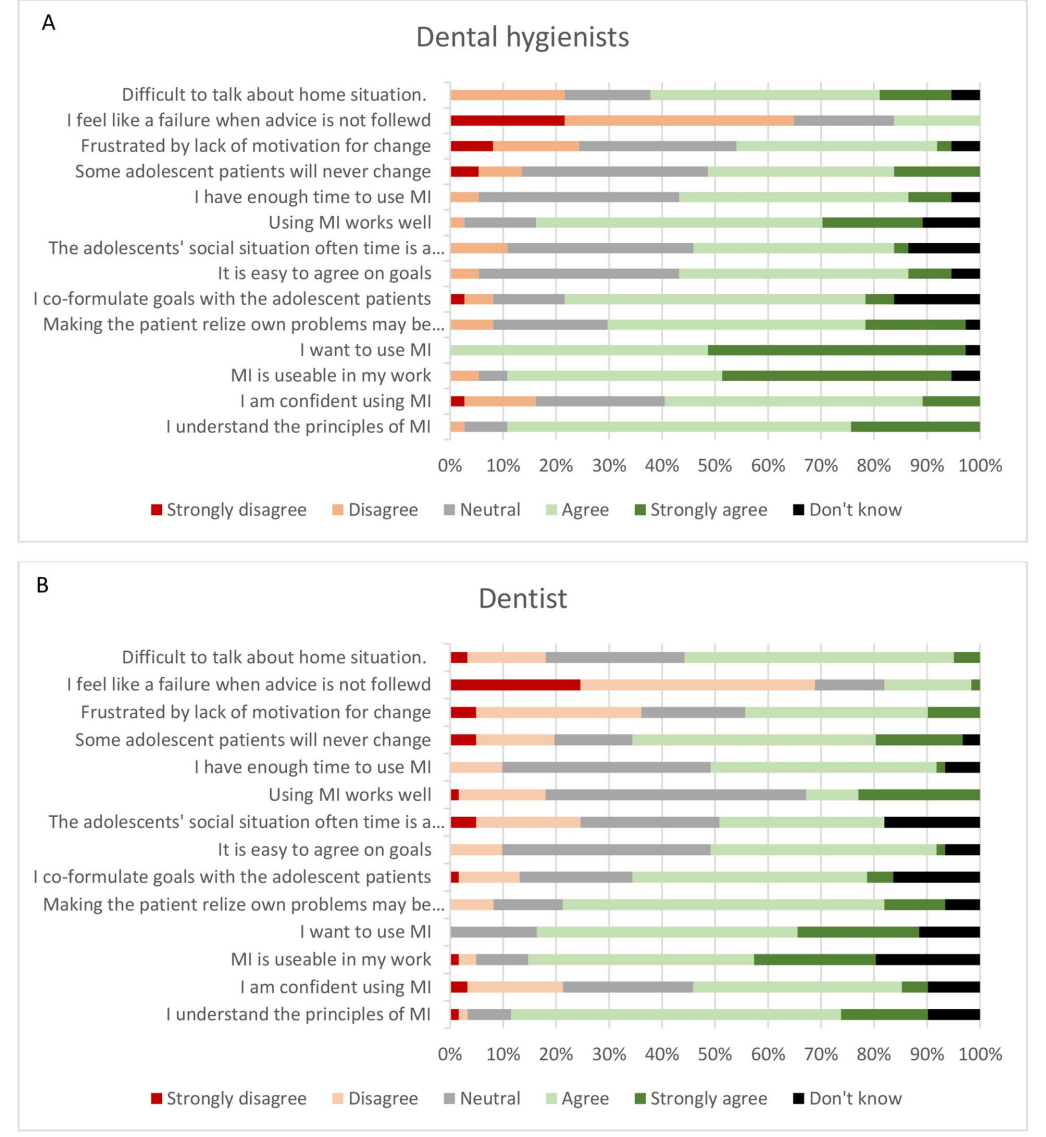
Dental professionals’ beliefs and attitudes regarding the use of MI for adolescent patients (**A**) dental hygienists’ responses; (**B**) dentists’ responses

Respondents were asked to consider various statements regarding the use of MI in dental practice. As can be seen in Fig. 1A and B, about 90% of dental hygienists and 80% of dentists felt that they understood the principles of MI (combining agree and strongly agree). However, only about 45% and 60%, respectively, felt confident in the use of MI. Overall, dental hygienists were more positive towards, and inclined to use MI in dental practice than was the case for dentists. In comparison to dentists, dental hygienists found MI more usable in their work (p = 0.052), want to use MI to a greater extent (p = 0.002), and find that using MI works well for them (p < 0.001).

About 45% of the dentists and 50% of the dental hygienists considered that they had enough time to use MI. Furthermore, about 50% of the dentists and 63% of the dental hygienists co-formulated goals with the adolescent patients. While around half the respondents confirmed that it is easy to agree on the goals with the adolescents, as much as 44% of dentists and 41% of dental hygienists are frustrated by the lack of motivation to change in some adolescents. More than half of the respondents found it difficult to discuss adolescent patients’ home situations and believed some adolescents will never change no matter what they do. 31% of the dentists and 41% of the dental hygienists agreed with the statement that the adolescents’ social situation often is a hindrance. Of the respondents, 18% of the dentists and 16% of the dental hygienists even felt like a failure professionally due to adolescents not complying with advice given.

From responses to the open questions in the questionnaire, there appeared to be inconsistencies regarding the use of MI in conversations with patients. Thirteen [13] of the dental personnel, 10 dentists and three dental hygienists, responded that they did not know if they used MI at work, which might be exemplified by this statement made by a dentist: “[I] Always try to motivate the patients to better oral hygiene, but unsure if I am using the MI method in the right way as I have never been trained in what it actually entails to use MI versus talking openly with the patient about what they are doing and my advice and recommendations.“

## Discussion

### Reflection on key findings

Although MI is embedded within undergraduate and postgraduate dental education in Norway, as well as being supported by national guidelines, this is the first study to explore how dental professionals use MI in everyday clinical practice with their adolescent patients. This study has also provided an important insight into the varying practices and attitudes of both dentists and dental hygienists. The implications of the key findings, for both educators and service providers will now be considered.

It is evident that dental professionals in Norway are committed to preventing oral diseases in children and adolescents, and some can find it frustrating when their advice does not result in improvements in oral health behaviours. Many of them have received training in MI and attempt to apply this intervention in the care of adolescent patients but do not all feel confident to use this approach. The availability of training and inclusion of MI in the national guidelines has not been sufficient to ensure that MI is implemented, and the reasons for this need to be explored.

As can be seen from the results, there is a paradox that while a large majority of participants indicate an understanding of the theoretical foundations of MI, only about half feel confident in its application. Some previous studies on the use of MI in healthcare have pointed to similar issues. For instance, the integrity of MI inventions, i.e., are they being performed consistently with regards to theory, has been questioned, and problems may be related to the fact that emphasis has been put on the “spirit of the method” rather than on teaching explicit competencies [33]. Also, while some of the theoretical concepts in MI may be easily understood and disseminated, which is reflected by the current results, the same concepts may be harder to measure or enact in practice. For instance, concepts such as egalitarianism and empathy might be defined, measured and enacted quite differently by different actors, which could create uncertainty about how to go about MI. If we consider that the participants of the current study indicated a substantial variation in the extent of their received MI training, which one would think could help “translate” theory into practice, the apparent disjunction between theory and practice is less than surprising. Luckily, studies have demonstrated that MI interventions can be taught successfully, and that there are approaches available to ensure that the integrity of MI is maintained through training [33]. A recent study from the dental setting also shows that the basic principles and skills of MI can be taught successfully by using relatively simple e-learning programmes [34], which could help disseminate the overall theoretical approach of MI to a larger audience.

Most respondents with training in MI reported receiving this at postgraduate education courses, but there is no information on what type of training was covered. A systematic review looking at training in MI [35], found that most studies failed to specify the type of training provided, general or specific MI training. If this applies to the training received by the respondents in the current study, it could explain why some of them were unsure about what differentiated MI from basic professional communication [36]. The national recommendations do not help provide a clear distinction between the two, as MI is recommended not only to be used as a tool for behaviour change but as an aid to facilitate good communication and building a professional relationship. The recommendations of use seem to be failing to describe MI as a particular tool for addressing specific problems. The complexity of the skill required, and recognition of the practice needed to develop and use it adequately have not been fully addressed, confusing the simplicity of MI with being easy to practise [10]. MI is described and intended for use across a variety of professions. Thus, the professional competencies will vary. Perhaps the reflections from participants in the current study indicate that the training in MI for dental health professionals could benefit from more focus on MI strategies rather than theory.

Further barriers to the implementation of MI may include time constraints or lack of MI interventions which have been designed for changing oral health behaviours in adolescents. Clearly, the specific characteristics of adolescents need to be understood for any health intervention to be efficacious; engagement and therapeutic alliance being crucial when working with this age group [16].

Another key finding from this study is that dental hygienists appear more likely to adopt MI as a tool for behaviour change when working with adolescents compared to general dentists. This is consistent with the work of Thevissen, De Bruyn [37] which also showed that hygienists were more frequent users of patient motivational interventions than their dentist colleagues. However, it must be acknowledged that the case mix and scope of practice of dental hygienists is quite different to that of dentists. It therefore cannot be inferred that dental hygienists have a more positive attitude towards MI, rather it may be that their patients are more suited to this intervention. Furthermore, a greater proportion of dental hygienists in this study reported having received training in MI, from both undergraduate and postgraduate/continuing education courses, compared to general dentists. This finding may also explain their greater adoption of MI, as maintaining this practice within routine patient care is challenging. Indeed, Leske, Mustchin [38] highlighted that MI is a skill that requires ongoing training, practice as well as mentoring to be effective. An RCT conducted by Lozano, McPhillips [39] demonstrated improved behaviour change counselling by paediatric trainees following 9-hours of structured training in delivering brief MI to parents of children with asthma. However, when participant’s performance was re-assessed 3- and 7-months after receipt of training, the authors acknowledged that skills decline without additional training and feedback sessions. A substantial proportion of dentists in Norway obtained their education in other countries. In 2021, 53% of authorizations were given to dentists educated abroad [40]. In the same year, only 9% of authorizations in dental hygiene were given to professionals educated outside of Norway. As mentioned, MI falls within the curriculum of all education in dentistry and dental hygiene offered in Norway. It is unclear whether, and to what degree, students studying outside of Norway are trained in MI.

### The use of MI with adolescents

Feedback from the respondents suggested that they perceived external conditions in some adolescents’ lives made it difficult for them to change their behaviours. While respondents expressed an understanding of the individual perspective and different opportunities to change, many also found it difficult to discuss such external conditions, potentially failing to address important barriers to improving adolescents’ oral health behaviours. Our findings imply that clinicians may feel ‘uncomfortable’ when discussing topics that could potentially raise difficult emotions in their young patients (i.e., regarding their home situation). Home environment plays an important role in building habits [41] and forming healthy behaviours in adolescents, and oral health practices. So understanding home situations becomes essential for dental health practitioners to help young people develop good dental health. Adolescents may feel uncomfortable to share information because they may feel it is a sensitive topic, or dental health personnel may feel uncomfortable to ask because they might not know how to address sensitive topics. This can be achieved by creating a welcoming and non-judgemental environment where adolescents feel comfortable sharing information. Dental personnel can also receive additional training on how to address sensitive topics such as this. The accepting and supporting therapeutic stance in MI demand the ability to regulate one’s own discomfort and negative emotions when facing challenging interpersonal behaviour from the patients [42]. Thus, enhancing and developing emotional competence in practitioners could prove beneficial.

It is unclear whether the notion that some adolescents never will change their behaviour, and the frustration expressed when adolescents fail to comply, is related to the experience of repeated relapse. Paradoxically, relapse is normalised in MI; however, based on the attitudes toward MI in this survey, relapse seems to be translated into failure in both the practitioners’ and patients’ capabilities, compromising rather than supporting self-efficacy [42]. It can be hypothesised that if MI were implemented with more rigour, the attitudes and feelings of failure would decrease, potentially increasing the use and benefits of MI.

The finding that more than half of dental health personnel believe that some adolescents will never change no matter what they do, is concerning. It suggests a potential sense of hopelessness among dental personnel about their ability to promote dental health in adolescents. While some adolescents might not change by the solution that they provided, they should not give up on them. Some adolescents may need unique strategies and tailored education or interventions in multiple steps and as a thread over a longer period. Promoting a sense of hope and hopefulness in both dental personnel and adolescents can be a powerful motivation for change and reaching the goal.

Considering that around half of the dental health personnel in this survey find it easy to agree on goals for improving adolescents’ oral health indicates a positive human-centred approach to dental care. This approach recognizes patients as active participants in their own health and the importance of involving them in decision-making processes. This involvement can lead to more successful behaviour change and improved dental health outcomes. Dental professionals can better comprehend patients’ needs and motivations, enabling them to tailor recommendations to suit individuals. This approach aligns with the principles of MI, which aim to explore and enhance an individual’s motivations.

### Strengths and limitations of the study

Several limitations are acknowledged with respect to the present study. Firstly, the survey was only completed by dental health professionals working in one region of Norway (Trøndelag PDS), which limits the generalisability of findings to the wider country, as well as to other differently funded or organised dental services. Furthermore, we do not know the working hours of each respondent to determine whether there were differences between the two professional groups and to what extent this could have impacted on their overall training in, experience with, and perception of using MI when working with adolescent patients. However, we have no reason to believe that this has introduced bias to our results. Additionally, although the questionnaire was subject to a pilot study, the final document was lengthy and may have presented a greater time burden to participants than anticipated.

Despite the length of the questionnaire, however, a favourable response rate (58.3%) was achieved, which is in line with the 60% or above that is recommended for this type of study [43]. This study also provides insight into the actual uptake of MI by dental professionals in a dental service which is generally supportive of this intervention. It is surprising that, although the literature supports the use of MI in dental practice, little is known about the views and practices of dental professionals who have received training in MI, particularly with adolescents.

### Future service development and research

Although this study has, for the first time, reported on the use of MI by dental professionals in Norway, further qualitative research is warranted to better understand why some dental health professionals are more likely to incorporate MI in their practice, than others. A greater insight into the nature of the MI encounters with adolescents would also be invaluable. It is suggested that there is a need for co-designed interventions to achieve oral health behaviour change in adolescents so that they better meet the needs of this patient group. The use of MI interventions specific to each behaviour, for example improving toothbrushing, reducing sugar consumption, or smoking cessation may prove to be more effective than a more ‘generic’ MI approach. Such tailored interventions may also better support clinicians in the delivery of MI. A further suggested development for the service would be that the delivery of MI is monitored by participating practices, using validated tools, to ensure that MI is being delivered as intended and to re-evaluate their own learning needs.

## Conclusions

This survey has shown that a relatively high proportion of dental professionals, working within a Norwegian public dental service, have reportedly received training in MI. However, some disparity exists between clinicians in terms of implementation. Although MI is an evidence-based approach for improving oral health behaviours, further research is indicated to better understand, and address, any barriers to its wider adoption for adolescent patients.

## Data Availability

The data used and analysed during the current study are available from the corresponding author on reasonable request.

## Abbreviations

MI: motivational interview
PDS: public dental service
